# The genome portal of the Department of Energy Joint Genome Institute: 2014 updates

**DOI:** 10.1093/nar/gkt1069

**Published:** 2013-11-12

**Authors:** Henrik Nordberg, Michael Cantor, Serge Dusheyko, Susan Hua, Alexander Poliakov, Igor Shabalov, Tatyana Smirnova, Igor V. Grigoriev, Inna Dubchak

**Affiliations:** Department of Energy Joint Genome Institute, Walnut Creek, CA 94598, USA

## Abstract

The U.S. Department of Energy (DOE) Joint Genome Institute (JGI), a national user facility, serves the diverse scientific community by providing integrated high-throughput sequencing and computational analysis to enable system-based scientific approaches in support of DOE missions related to clean energy generation and environmental characterization. The JGI Genome Portal (http://genome.jgi.doe.gov) provides unified access to all JGI genomic databases and analytical tools. The JGI maintains extensive data management systems and specialized analytical capabilities to manage and interpret complex genomic data. A user can search, download and explore multiple data sets available for all DOE JGI sequencing projects including their status, assemblies and annotations of sequenced genomes. Here we describe major updates of the Genome Portal in the past 2 years with a specific emphasis on efficient handling of the rapidly growing amount of diverse genomic data accumulated in JGI.

## INTRODUCTION

A leader in genome sequencing, the DOE JGI has significantly expanded its capabilities to achieve a deeper understanding of biological functions encoded by DNA. With the increasingly diverse data types it generates and associated computational strategies for their analysis, the DOE JGI is focused on enabling its community of users to make important new scientific discoveries that address major energy and environmental challenges (U.S. Department of Energy Joint Genome Institute Progress Report, http://www.jgi.doe.gov/whoweare/JGI-progress-report-2012.pdf).

In 2012, JGI completed 2635 projects, a 3-fold increase over 2011, and generated >56 trillion nucleotides of genome-sequence data from microbes and microbial communities, fungi, algae and plants. In the past year alone, JGI has added 650 genomes to the public databases. JGI sequencing efforts are mostly based on the proposals submitted to JGI by researchers and to a large extent are aligned with several large-scale JGI programs such as Community Science Program (CSP) and Genomic Encyclopedia of Bacteria and Archaea (GEBA) (1). The amount of data being produced by sequencing and analyzing genomes brings genomics into the realm of big data category. The increased scale in data has demanded a corresponding advance in the scale and robustness of the computing infrastructure underlying the Genome Portal. Since the previous publication on the JGI Genome Portal in January 2012 (2), we specifically focused on improvement of computational resources for efficient storage, access, download and analysis of these data.

## DATABASES ACCESSIBLE THROUGH INTEGRATED GENOME PORTAL

**Figure 1.**
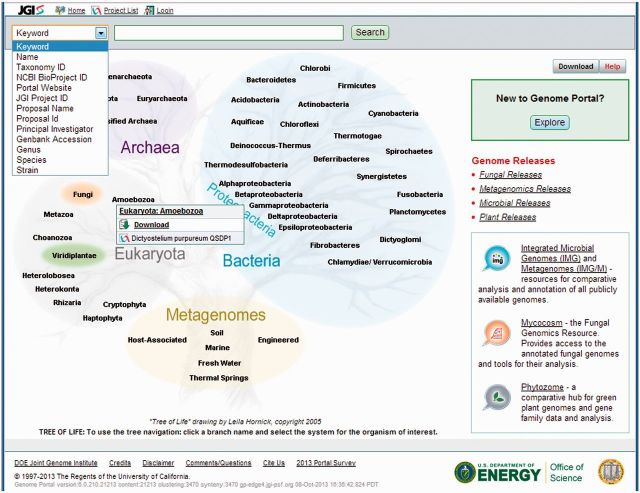
The Genome Portal page. Pull-down menus for the Search categories and the Amoebozoa branch of Eukaryota are shown.

The Portal home page, available at http://genome.jgi.doe.gov, allows a user to select the genomic data set and the tools to work with (Figure 1). It provides the information about the latest genome releases and access to several specialized JGI database resources: integrated microbial genomes (IMG) (3) and metagenomes (IMG/M) (4), Phytozome for green plant genomes (5) and MycoCosm for fungal genomes (2). It also provides access to extensive help pages as well as a recently developed interactive tutorial for novice users.

There are two major categories of data available in JGI sequencing projects and annotated genomes and metagenomes. More than 15 000 DOE JGI projects of different types are publicly available and searchable in our database. These projects include different genomic products, such as standard and improved draft, finished genomes, gene expression profiling, resequencing, metagenome projects, single cell projects, transcriptomes, metatranscriptomes, exomes, specialized analysis types and others. The Genome Portal provides access to over 17 000 annotated genomes and metagenomes available in the DOE JGI database, along with specialized analytical tools to navigate these data sets and compare genomes.

## DATA ORGANIZATION AND ACCESS

**Figure 2.**
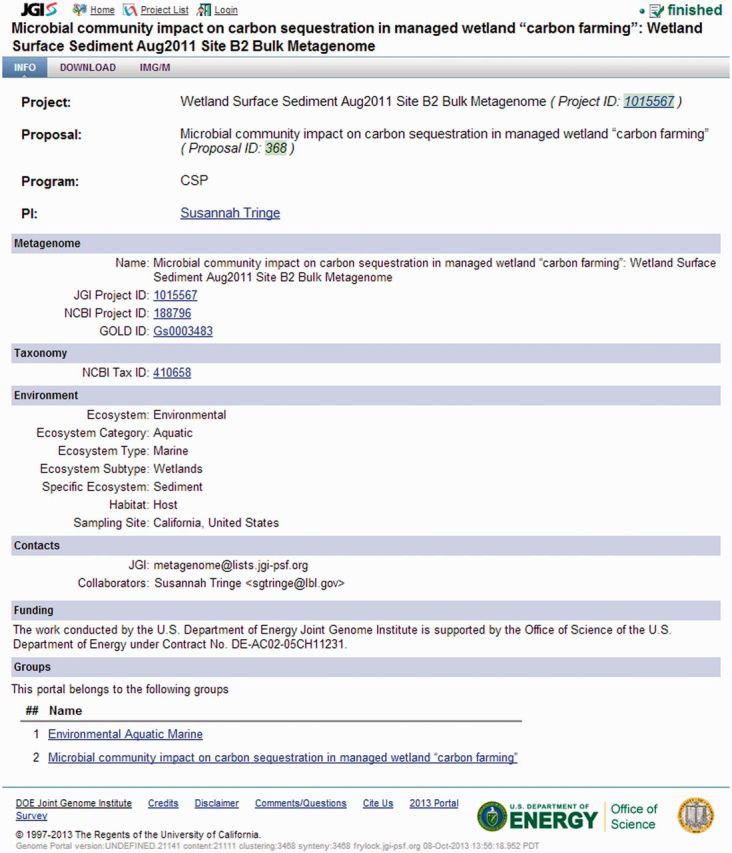
Individual portal page for the Wetland Surface Sediment Metagenome project. Relevant information is classified into several sections including ‘Groups’ that specifies an appropriate proposal or large-scale JGI programs.

Individual Genome Portal project sites (‘portals’) are created automatically on passage of sample quality control stage for a given sequencing project. The JGI has ∼24 000 portals for isolate, metagenome, single-cell, transcriptome and resequencing projects. Portals aggregate relevant analysis tools for each project and provide download access to project data and project information such as proposal name, organism and taxonomy info as well as the names of principal investigator and other contacts relevant to an associated data set (Figure 2).

To navigate the large volume and diversity of genome projects at the JGI, the Genome Portal offers several new and improved tools to locate a genome of interest: a list of all JGI projects, a ‘Search’ function, an interactive ‘Tree of Life’ and domain-specific comparative resources. Data access and the spectrum of data depend on the stage of each project and the role of users in these projects (e.g. administrative). Free registration allows users not only to access all data but also to initiate new projects with JGI.

The ‘Project List’ link on the Genome Portal page (http://genome.jgi.doe.gov) (Figure 1) brings users to a list of all DOE JGI projects with a detailed description of each project including its scope and current status, taxon, the JGI program, the project lead and the computational resources available for this project.

The JGI Genome Portal now includes a new Portal Search function that enables searching for genomes and projects by keyword (e.g. plants, algae, single cell, water), name and other categories of data (shown in a pull-down menu, Figure 1). The user can also locate projects related to specific classifications such as a proposal or a project. Typing the beginning of the word in the text window brings up a pull-down menu with relevant search term choices.

**Figure 3.**
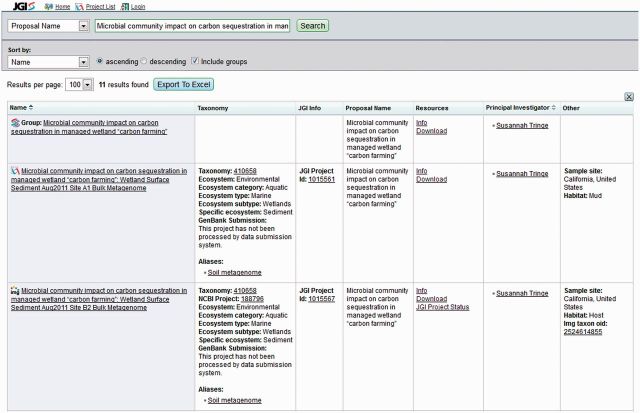
Search results table. ‘Info’ link in the Resources column leads to an individual portal of each found data set.

Search results now include Project information and status, GenBank accession, taxonomic information, PI contact and much more (Figure 3). Every JGI Proposal, which can include multiple genomes, transcriptomes or other data sets, now has all related portals grouped together, and downloads of entire segments of data are possible at the proposal level.

The Tree of Life organizes the sequenced genomes by the domains of life and metagenomes by ecological ‘niche’. Clicking on a branch name produces a menu displaying available genomes in the corresponding kingdom, phylum, class or order. Selecting an entire group of genomes or a particular genome connects a user to a corresponding organism page or pages in specialized resources indicated by associated icons (Figure 1). Microbial, archaeal genomes and metagenomes are directed to the corresponding IMG and IMG/M pages, fungal genomes to Mycocosm and plant genomes to Phytozome. Genomes not included in either of these specialized resources are linked to their individual portal pages containing information about the corresponding project and available tools.

## INTRASTRUCTURE

The Genome Portal Web site is built on Apache HTTPD, Tomcat and MySQL. A majority of the Genome Portal components have been developed using Java and a variety of available open-sources tools and technologies. There are four load-balanced web servers, talking to two back-end database servers. A web-driven automated build system that uses Jenkins (http://jenkins-ci.org/) takes each machine silently out of the cluster, builds a new version of the portal and puts the machine back into the cluster. This procedure ensures that updates can be applied without disruption to users. This setup further makes the portal resilient against hardware failures.

The Genome Portal acquires data from several of JGI’s annotation pipelines via an API and it makes the data available to authorized users without delays. There is an advanced monitoring system in place that allows preventing problems that may impact Web site and database performance.

Over the last 2 years, we have forged a strong alliance in high-performance computing with the National Energy Research Scientific Computing Center (NERSC). NERSC now hosts the servers on which the Genome Portal is running and provides access to ESnet (Energy Sciences Network), which allows for top of the class transfer rates.

## DATA DOWNLOAD

The Genome Portal currently supports download of multiple file types: all raw and quality control data, assemblies and various analysis files. All files can be accessed in a variety of ways, each designed with a particular class of users in mind.

**Figure 4.**
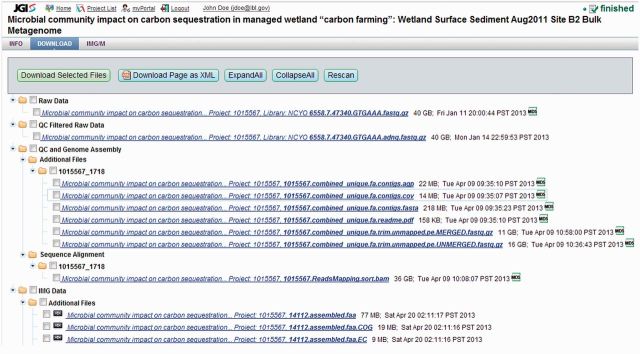
Download function access page for the Wetland Surface Sediment Metagenome project. A user can select any relevant data set/s of raw data, genome assembly and analysis results.

In the web-driven approach to data access, the user finds a portal of interest and clicks on the download tab of that portal. Downloads are available in a tree structure that divides the files into logical groups so that the user can download raw data files, assemblies and so on with a single operation (Figure 4).

For users who need to download a large number of files or large files (with the largest being several hundred gigabytes), data are available via the public JGI endpoint on GlobusOnline (https://www.globusonline.org/). This requires that the user log on to globusonline.org and activate the ‘jgi#portal’ endpoint using their JGI credentials. GlobusOnline then provides a user-friendly interface for facilitating big data transfers. Behind the scenes the transfers are performed using GridFTP (6), which is a parallel transfer protocol and program. GridFTP has built-in checks that ensure the integrity of the transfers and guarantees that the files reach their destination intact.

Finally, a web service is available for users who seek to download JGI data programmatically. This service is described in the documentation on the Genome Portal Web site.

Whether a user navigates the web user interface, uses GlobusOnline or uses the web service, the data that can be accessed are the same in each case. As the amount of data the JGI makes available is counted in petabytes, many of the files are stored on the NERSC HPSS mass storage archive. All three approaches hide this fact from the user, interacting with the tape system to cache files as required.

In addition to downloading data, The Department of Energy Systems Biology Knowledgebase (KBase) (http://kbase.science.energy.gov/) users can upload their data directly from the portal page to KBase. They do so by selecting files in the tree on the download page, clicking ‘Push to KBase’ and entering their KBase credentials.

## DATA SUBMISSION TO NCBI

The increasing volume of genome data has led the NCBI to develop a new automatic submission system (7) to streamline the process of submitting complex high-throughput sequencing or functional genomic data sets. The DOE JGI has collaborated with the NCBI to develop the first major implementation for a sequencing center of a framework to use this submission system. Using this system, the JGI Genome Portal automatically generates and monitors BioSample and BioProject submissions to NCBI using an XML-based submission protocol. Internally, we use the same infrastructure to prepare GenBank submissions. The latter are submitted to the NCBI FTP server using the ASN.1 and related GenBank-specific formats.

## References

[1] Wu D, Hugenholtz P, Mavromatis K, Pukall R, Dalin E, Ivanova NN, Kunin V, Goodwin L, Wu M, Tindall BJ (2009). A phylogeny-driven genomic encyclopaedia of bacteria and archaea. Nature.

[2] Grigoriev IV, Nordberg H, Shabalov I, Aerts A, Cantor M, Goodstein D, Kuo A, Minovitsky S, Nikitin R, Ohm RA (2012). The genome portal of the department of energy joint genome institute. Nucleic Acids Res..

[3] Markowitz VM, Chen IM, Palaniappan K, Chu K, Szeto E, Grechkin Y, Ratner A, Jacob B, Huang J, Williams P (2012). IMG: the integrated microbial genomes database and comparative analysis system. Nucleic Acids Res..

[4] Markowitz VM, Chen IM, Chu K, Szeto E, Palaniappan K, Grechkin Y, Ratner A, Jacob B, Pati A, Huntemann M (2012). IMG/M: the integrated metagenome data management and comparative analysis system. Nucleic Acids Res..

[5] Goodstein DM, Shu S, Howson R, Neupane R, Hayes RD, Fazo J, Mitros T, Dirks W, Hellsten U, Putnam N (2012). Phytozome: a comparative platform for green plant genomics. Nucleic Acids Res..

[6] Allcock W, Bresnahan J, Kettimuthu R, Link M, Dumitrescu C, Raicu I, Foster I (2005). The Globus striped GridFTP framework and server. Proceedings of the 2005 ACM/IEEE Conference on Supercomputing.

[7] NCBI Resource Coordinators (2013). Database resources of the national center for biotechnology information. Nucleic Acids Res..

